# Inhibition of Prostate Cancer Cells by 4,5-Dicaffeoylquinic Acid through Cell Cycle Arrest

**DOI:** 10.1155/2019/4520645

**Published:** 2019-05-23

**Authors:** Olivia Lodise, Ketki Patil, Igor Karshenboym, Scott Prombo, Chidinma Chukwueke, S. Balakrishna Pai

**Affiliations:** Wallace H. Coulter Department of Biomedical Engineering, Georgia Institute of Technology and Emory University, 313 Ferst Drive, Atlanta, Georgia 30332, USA

## Abstract

Prostate cancer is a major cause of cancer-related mortality in men. Even though current therapeutic management has contributed to reducing mortality, additional intervention strategies are warranted to further improve the outcomes. To this end, we have investigated the efficacy of dicaffeoylquinic acids, ingredients in Yerba Mate (*Ilex paraguariensis*), an evergreen cultivated in South America, the leaves of which are used to prepare a tea/coffee-like drink. Of the various analogs tested, 4,5-dicaffeoylquinic acid (4,5-diCQA) was the most active molecule against DU-145 prostate cancer cells with a 50% inhibitory concentration (IC_50_) of 5 *μ*M. 4,5-diCQA was active both under normoxic and hypoxic conditions. The effect of 72-hour treatment on DU-145 cells persisted for an extended time period as assessed by clonogenic assay. Mechanistic studies revealed that the toxic effect was not due to induction of programmed cell death but through cell cycle arrest at S phase. Additionally, 4,5-diCQA did not impact PI3K/MAPK signaling pathway nor did it affect the depolarization of the mitochondrial membrane. 4,5-diCQA-induced accumulation of cells in the S-phase also seems to negatively impact Bcl-2 expression. 4,5-diCQA also exhibited inhibitory activity on LNCaP and PC-3 prostate cancer cells suggesting that it has therapeutic potential on a broad range of prostate cancers. Taken together, the novel inhibitory activity and mechanism of action of 4,5-diCQA opens up potential therapeutic options for using this molecule as monotherapy as well as in combinatorial therapies for the clinical management of prostate cancer.

## 1. Introduction

Global cancer statistics of mortality indicate that 9.6 million people died of cancer in 2018 [1]. A World Cancer Report estimated prostate cancer incidence of 31.1 in 100,000, next only to lung cancer which is the number one cancer in men (34.1 in 100,000) [2]. Further, estimates of age-standardized incidence for cancers for both sexes for the year 2018 rank prostate cancer second after breast cancer, whereas similar standardized data for men show prostate cancer a close second to lung cancer [3]. Therefore, more effective treatments are warranted to decrease the high mortality rate. Molecules with anticancer activity from natural sources are of particular interest lately as they often have less harmful side effects compared to conventional chemotherapy. Also, they can be potentially combined with current cancer treatments to increase their effectiveness against prostate cancer while also mitigating side effects.

Yerba Mate (YM) (*Ilex paraguariensis*), which is used to prepare a tea/coffee-like drink in South America, has been shown to contain many biologically active chemicals with a range of health benefits [4–6]. Some of the major constituents of this drink, namely, polyphenols and dicaffeoylquinic acids (diCQAs), have been investigated for their biological activity. Whereas several studies have focused on the antioxidant and anti-inflammatory properties of YM extracts containing diCQAs, their antineoplastic effects have not been extensively studied. These chemicals have only been studied for their anticancer properties primarily against a few cancers including gastrointestinal cancers because of the oral consumption of the product [7, 8]. Further, the YM components also demonstrated activity against pancreatic lipases [9]. Detailed studies on the anticancer effects and mode of action of this class of compounds are lacking.

The present study was undertaken to investigate the efficacy of diCQAs on prostate cancer cells. Using prostate cancer cell lines such as DU-145, LNCaP, and PC-3, we explored the efficacy of diCQAs to inhibit cell proliferation. Further, we elucidated the underlying mechanism of 4,5-diCQA-induced cell death using the highly metastatic prostate cancer cell line DU-145.

## 2. Materials and Methods

### 2.1. Cell Lines and Materials

Cell lines used were obtained from American Type Culture Collection (ATCC). Complete growth medium for DU-145 cell line was prepared by supplementing Eagle's Minimum Essential Medium with 10% Fetal Bovine Serum (FBS), 1% Penicillin/Streptomycin, and 2 mM L-Glutamine. Cultures were incubated at 37°C in atmosphere of 5% CO_2_ and 95% air. 3,4-diCQA, 3,5-diCQA, and 4,5-diCQA were all purchased from Sigma (SMB00224, SMB00131, and SMB00221, respectively) and dissolved in DMSO to create the stock solutions.

### 2.2. Cytotoxicity Assessment of diCQAs on DU-145

DU-145 cells were plated in 96-well plates (5000 cells/well) in triplicate for the control and diCQA treatments. After 24 hours of incubation, medium was replaced with either fresh medium (for the control) or media with various concentrations of the compounds. Concentrations used for 4,5-diCQA were 100 *μ*M, 75 *μ*M, 50 *μ*M, 25 *μ*M, 10 *μ*M, 5 *μ*M, 1 *μ*M, and 0.1 *μ*M while concentrations tested for 3,4-diCQA and 3,5-diCQA were 100 *μ*M, 75 *μ*M, 50 *μ*M, 25 *μ*M, and 10 *μ*M. The cells were incubated for 72 hours. To generate hypoxic condition, 100 *μ*M of Cobalt chloride was added to the complete growth medium. After 72 hours of treatment period, cell viability was assessed by performing a Cell Counting Kit-8 (CCK-8) assay as per the manufacturer's protocol (Bimake CCK-8 protocol B34305).

### 2.3. Clonogenic Assays

Cells (100,000/well) were treated in triplicate for 72 hours with 5 *μ*M of 4,5-diCQA in 6-well plates. After the treatment period, cells were collected by trypsinization and counted. For determining the colony forming efficiency of the cells, control and treated cells were seeded in 6-well plates at density of 500 cells per well and incubated in drug-free media and left to proliferate for 9 days. For visualization of the colonies formed, the culture media were removed followed by washing the cultures with PBS, fixing with ice-cold methanol, and then staining with crystal violet solution.

### 2.4. MUSE® Flow Cytometric Analysis

For the various flow cytometric analyses, 1 × 10^5^ cells per well were seeded in triplicate in 6-well cell culture plates and treated with 5 *μ*M of 4,5-diCQA for 72 hours. Cells were then harvested and stained as per the MUSE cell kit protocols. The various flow cytometric analyses performed are MUSE Annexin V & Dead cell assay (MCH100105), Mitopotential assay (MCH100110), Bcl2 Activation Dual detection assay (MCH200105), PI3K-MAPK Dual Activation detection assay (MCH200108), and Cell Cycle analysis (MCH100106) (EMD Millipore). The cells were analyzed as per the manufacturer's instructions provided in the kit (assay kit reference numbers provided in parenthesis above) using a MUSE cell analyzer. Each experiment was done at least 3 times independently.

### 2.5. Inhibitory Potential of 4,5-diCQA on LNCaP and PC-3 Cell Lines

To investigate whether 4,5-diCQA has inhibitory activity on other prostate cancer cells, toxicity studies were performed on LNCaP and PC-3 cell lines. Cells (5000/well) were seeded in 96-well plates in RPMI medium supplemented with 10% fetal bovine serum, 1% Penicillin/Streptomycin, and 2 mM L-Glutamine. After 24 hours, media were removed and adherent cells were treated with varying concentrations of 4,5-diCQA. To assess the toxicity, CCK-8 assay was performed after 72 hours.

### 2.6. Assessment of 4,5-diCQA Toxicity in Cancer Cells of Varied Tissue Origin

To determine the degree of anticancer activity of 4,5-diCQA on cancer cell lines other than prostate cancer, we used human colorectal adenocarcinoma cell line (DLD-1), human esophageal adenocarcinoma cell line (OE-33), human liver-derived adenocarcinoma cell line (SK-HEP-1), and human glioblastoma cell line (U87EGFP, with green fluorescent protein expression). Cells were seeded in 96-well plates at a density of 5000 cells/well. For OE-33 and DLD-1 cells, RPMI medium supplemented with 10% FBS, 1% Penicillin/Streptomycin, and 2 mM L-Glutamine was used. For U87EGFP and SK-HEP-1, DMEM and EMEM with similar supplements, respectively, were used. Cells were treated with 5*μ*M concentration of 4,5-diCQA for 72 hours after which period, CCK-8 assay was performed to assess cell viability. DU-145 cells were also treated at the same concentration. To assess the effect on nontumorigenic cells, NIH-3T3 mouse fibroblasts were cultured in DMEM with 10% FBS, 1% Penicillin/Streptomycin, and 2 mM L-Glutamine and MC-3T3 mouse preosteoblast cells cultured in *α*-MEM with 10% FBS were included in the above experiment.

## 3. Results

### 3.1. 4,5-diCQA Induces Toxicity in DU-145 Cells

**Figure 1:**
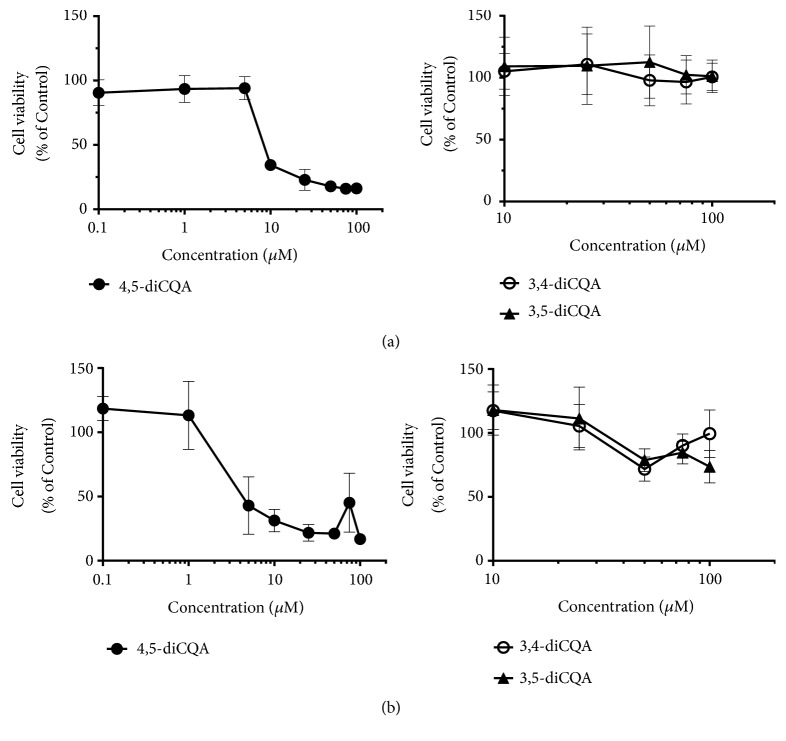
*Cytotoxicity profile of diCQA analogs on DU-145 prostate cancer cells*. (a) Cytotoxicity of 3,4-diCQA, 3,5-diCQA, and 4,5-diCQA on DU-145 cells in normoxic conditions was determined using CCK-8 assay. Mean values ± standard deviations from 8 trials are represented. (b) The cytotoxic effects of 3,4-diCQA, 3,5-diCQA, and 4,5-diCQA on DU-145 cells in hypoxic conditions were determined via CCK-8 assay. The means of the 8 trials were graphed along with their standard deviations.

To assess the anticancer potential of diCQA in human prostate cancer, DU-145 cells were treated with varying concentrations of three diCQA analogs (3,4-diCQA, 3,5-diCQA, and 4,5-diCQA) for 72 hours under both normoxic and hypoxic conditions. Treatment with 4,5-diCQA produced dose-dependent inhibition with an IC_50_ of 5 *μ*M. In comparison, 3,4-diCQA and 3,5-diCQA did not elicit toxicity to DU-145 cells even at high concentrations (Figure 1(a)). Therefore, 4,5-diCQA treatment was more effective than its isomers with a* p*-value = 0.00022. The same treatment was repeated for DU-145 cells grown under hypoxic condition generated by addition of 100 *μ*M CoCl_2_ (as described in “Materials and Methods”), and a similar dose dependent inhibition was observed (Figure 1(b)), with a p-value = 0.00776. The CCK-8 assay results demonstrated that 4,5-diCQA induces toxicity in DU-145 cells in a dose-dependent manner in both normoxic and hypoxic conditions, while other isomers of the compound are much less inhibitory.

### 3.2. 4,5-diCQA Inhibits the Proliferation and Colony Growth of DU-145 Cells after Treatment

**Figure 2:**
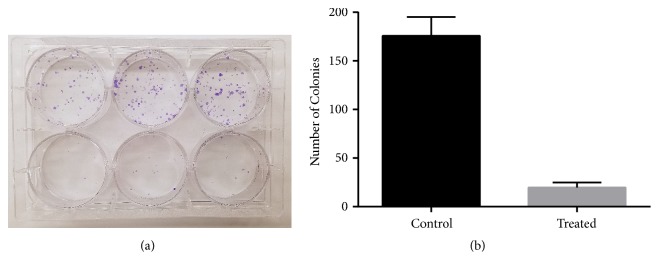
*4,5-diCQA decreases DU-145 cell proliferation after withdrawal of treatment*. Clonogenic assays were performed to determine the long-term effects of 4,5-diCQA on DU-145 cells. The treated cells were left to proliferate for 9 days in drug-free medium. (a) Representative image of the fixed and stained clonogenic assays showing reduced colony forming efficiency of the treated cells on the bottom row as compared to control on top row. (b) Colonies were counted and mean values of four trials ± standard deviations are represented. The treated group had significantly less colonies than the control (*p*-value = 5.64E-06).

Clonogenic assays were conducted to evaluate DU-145 cell proliferation and colony growth after treatment. The results demonstrated that 4,5-diCQA treatment for 72 hours impacted the ability of the cells to form colonies, resulting in fewer colonies in the treatment groups as compared with the control (Figures 2(a) and 2(b)). This suggested that the cellular effects induced by the 72-hour treatment continued to impact the cellular proliferation in the absence of 4,5-diCQA.

### 3.3. 4,5-diCQA Induces Cell Cycle Arrest in DU-145 Cells

**Figure 3:**
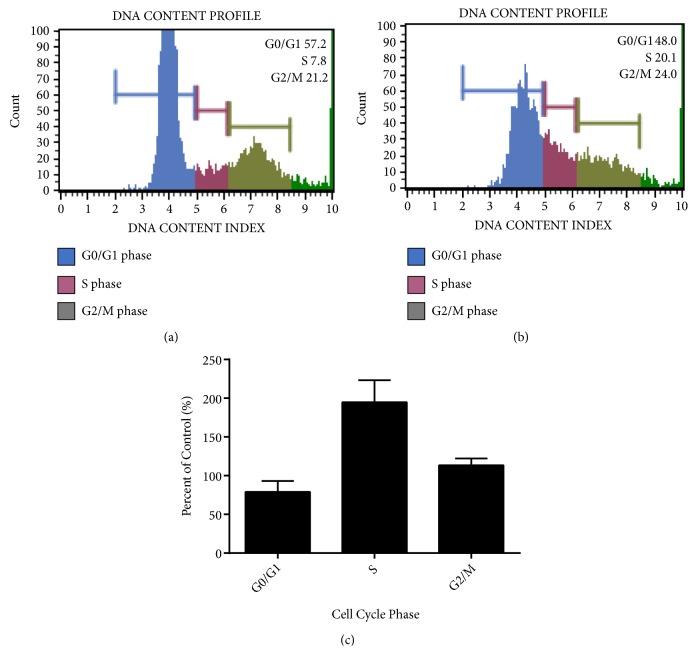
*Induction of cell cycle arrest in DU-145 cells by 4,5-diCQA*. Cell cycle analysis was performed by setting a threshold for live population based on cell size index and followed by setting gates for the cell cycle based on DNA content. Control group profile (a) was compared to the 4,5-diCQA group treated at the IC_50 _concentration (b). Majority (about 50%) of the treated cells were in the G0/G1 phase. (c) The percentage of cells in G0/G1 phase decreased (*p*-value = 0.3717), those in the S phase increased (*p*-value = 0.0075), and those in G2/M phase did not change significantly (*p*-value = 0.1726) compared with the control. Data represented are mean ± SD of four trials.

To determine the mechanism of action of 4,5-diCQA inhibition of DU-145 cell proliferation, MUSE flow cytometric cell cycle analysis was performed. Cells (1x 10^5^ cells/well) were plated and treated in 6-well plates with 5 *μ*M of 4,5-diCQA and compared with the control group (no treatment). The results showed that the cells treated with 4,5-diCQA decreased in numbers in G0/G1 phase and increased in S phase compared with the gating set for the control group (Figures 3(a) and 3(b)). The 4,5-diCQA treatment had twice the number of cells blocked in S-phase when normalized to control gated cells in S-phase (control S-phase population being considered as 100%) (Figure 3(c)).

### 3.4. 4,5-diCQA-Induced Toxicity Is Not Mediated through Programmed Cell Death in DU-145 Cells

**Figure 4:**
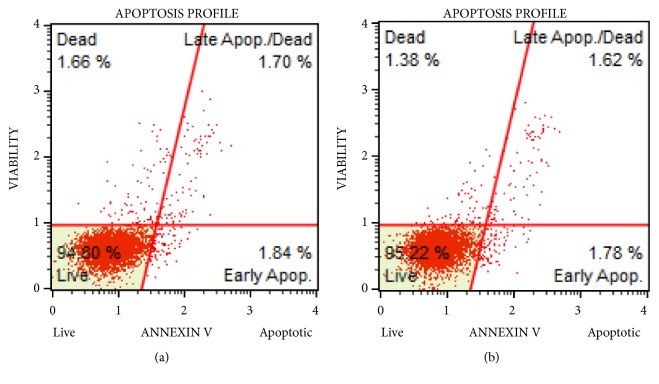
*Annexin V Assay confirms diCQA does not induce apoptosis in DU-145 cells*. The MUSE Annexin assay for the control cells (a) and cells treated with 5 *µ*M (IC_50_) of 4,5-diCQA for 72 hours (b).

**Figure 5:**
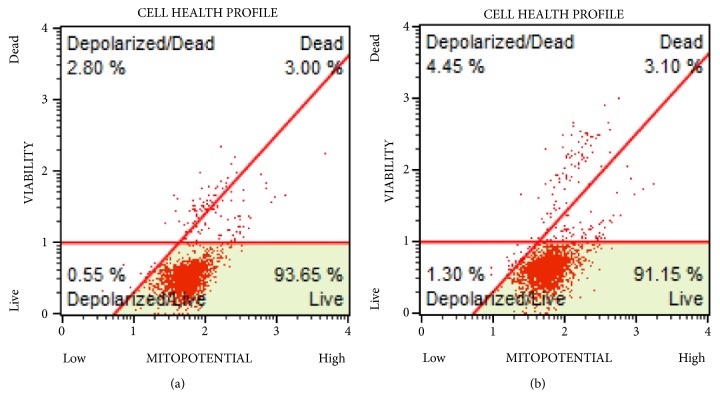
*4,5-diCQA does not induce depolarization of mitochondrial membrane potential in DU-145 cells*. The MUSE Mitopotential assay for the control (a) and the treated (b) was compared. The treated group consisted of cells with 5 *μ*M (IC_50_) of 4,5-diCQA for 72 hours, after which both groups were analyzed using a MUSE flow cytometer.

In an attempt to elucidate the mode of action of 4,5-diCQA, studies were undertaken to determine if the DU-145 cell death was due to induction of extrinsic or intrinsic apoptotic pathways. To this end, Annexin V MUSE flow cytometric assay was performed to compare cells treated with the IC_50_ concentration for 72 hours with the control group. The profiles of control and treatment groups were similar with no early or late apoptotic cells in the treatment groups ruling out the involvement of extrinsic apoptotic pathway (Figures 4(a) and 4(b)). Data from studies on analysis of depolarization of mitochondrial membrane showed no differences between control and treatment groups, suggesting that 4,5-diCQA does not exert its action through the intrinsic apoptotic pathway either (Figures 5(a) and 5(b)).

### 3.5. 4,5-diCQA Treatment Impacts the Bcl-2 Expression

**Figure 6:**
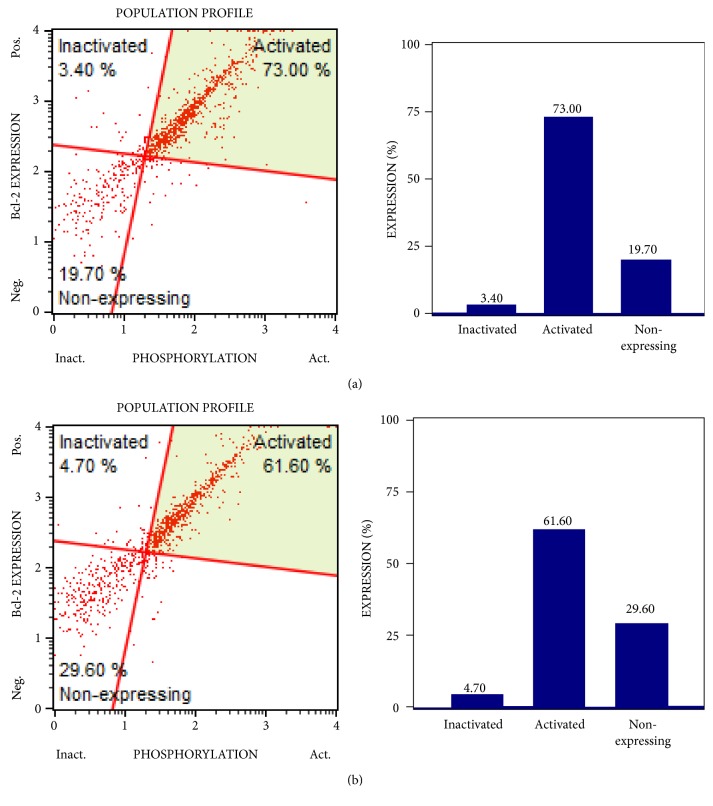
*Bcl-2 activation and expression profile of 4,5-diCQA-treated DU-145 cells*. MUSE Bcl-2 flow cytometric analysis for the control (a) and the 4,5-diCQA treated cells (b). 4,5-diCQA treatments were performed at IC_50_ concentration (5 *μ*M) for 72 hours, after which cells from control and treatment groups were collected, stained, and analyzed using a MUSE flow cytometer.

Since Bcl-2 expression plays a major role in survival of various cancers, we investigated whether 4,5-diCQA affected the Bcl-2 expression in the DU-145 cells. The 4,5-diCQA treatment showed that the compound induced inactivation of Bcl-2 with higher percentage of nonexpressing cells (Figure 6).

### 3.6. 4,5-diCQA Exhibits Inhibitory Activity on LNCaP and PC-3 Cell Lines

**Figure 7:**
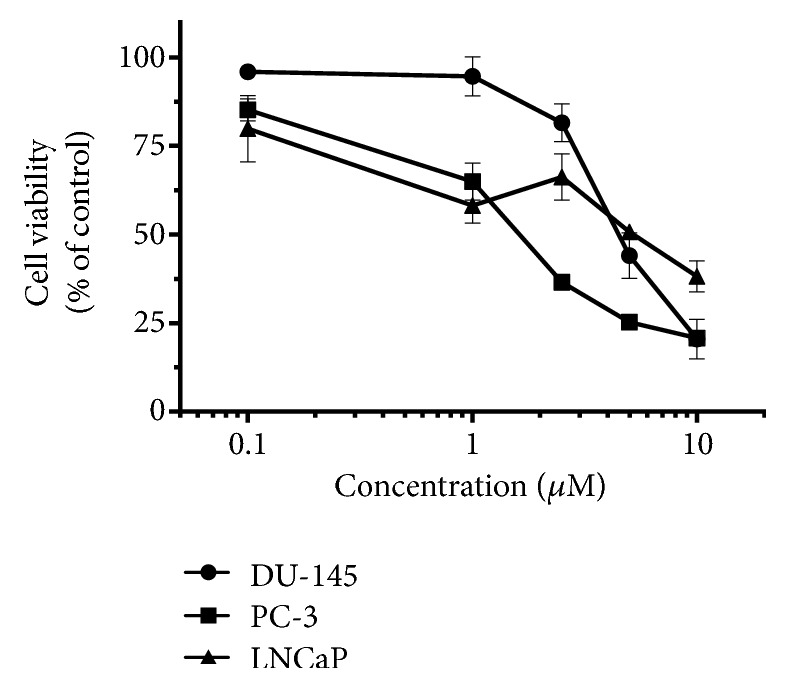
*Relative toxicities of 4,5-diCQA on DU-145, LNCaP and PC-3 prostate cancer cells*. Experiments were performed by seeding 5000/cells per well in 96-well tissue culture plate. After 24 hours, the culture medium was replaced with media containing 4,5-diCQA for treatment samples. Concentrations of 0.1 *μ*M, 1.0 *μ*M, 2.5 *μ*M, 5.0 *μ*M, and 10.0 *μ*M of 4,5-diCQA were used along with cultures with no treatments (control). After incubation for 72 hours, CCK-8 assay was performed by adding 10% CCK-8 solution in medium followed by incubation at 37°C after which absorbance was measured at 450 nm. Absorbances were normalized to untreated controls to determine cell viability. Mean ± standard deviation of triplicates is represented.

Cell viability assays performed after treatment of LNCaP and PC-3 prostate cancer cells revealed that 4,5-diCQA impacted the proliferation of these cells. Dose-dependent inhibition of LNCaP and PC-3 cells was observed. The toxicities on these cell lines were similar to that observed on DU-145. Further, PC-3 cells showed marginally higher sensitivity to 4,5-diCQA (Figure 7).

### 3.7. DU-145 Cells Were Most Sensitive to 4,5-diCQA in Comparison to Cancer Cells of Varied Tissue Origin

**Figure 8:**
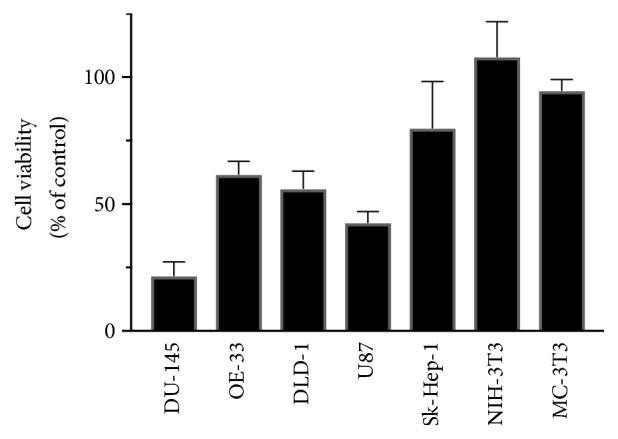
*Differential sensitivity of cancer cell lines and nontumorigenic cell lines to 4,5-diCQA*. The cell lines were cultured in their respective media as described in Section 2.6. Cultures were dissociated using trypsin and cells were plated in 96-well plates (5000 cells/well). After 24 hours, cells were treated with 5 *μ*M concentration of 4,5-diCQA in the growth medium. After 72 hours of treatment, cell viability was ascertained by the CCK-8 assay. Untreated cultures served as controls. Mean ± standard deviation of triplicates is represented.

Results from the studies on sensitivity of various cancer cell lines to 4,5-diCQA revealed that this molecule had maximal inhibitory activity on DU-145 cells. The inhibitory activity of 4,5-diCQA for the various cancer cell lines was DU-145 > U87 > DLD-1= OE-33 > SK-HEP-1. Moreover, at the concentration tested, 4,5-diCQA did not have any inhibitory activity on the noncancerous cell lines NIH-3T3 and MC-3T3 (Figure 8).

## 4. Discussion

Even though clinical management of cancer using chemotherapeutic agents has produced some success, there still exists a need to develop more effective treatment options. This necessitates the discovery and development of novel anticancer molecules. One of the current areas of investigation in this regard has been exploring the potential of active anticancer ingredients from natural products. These derivatives not only inhibit cancer cell proliferation, but also potentially have less side effects as compared to the currently used chemotherapeutics.

YM, the leaves of which are used in a popular drink in South America, has been suggested to have several health benefits. Multiple studies have focused on the biological activity of the YM extracts. Cuelho et al., in mice studies, observed chemoprotective effect of YM extract containing polyphenols on UVB exposure [10]. YM consumption also lowered the total blood cholesterol in subjects with dyslipidemia [11]. Further, administration of YM to rats had positive outcomes with regard to inflammation [12]. A majority of the studies were done with extracts of YM which contain a number of constituents including several polyphenols. Therefore, it is difficult to assign the observed activity to a specific molecule(s). This prompted us to test the anticancer potential of some of the ingredients such as diCQAs in their pure forms.

Our findings, for the first time, demonstrate the potent anticancer activity of 4,5-diCQA against prostate cancer cells. Additionally, clonogenic assay of cells treated with IC_50_ concentration for 72 hours and cultured in the absence of 4,5-diCQA showed that the inhibitory activity on cellular proliferation was sustained resulting in only twelve percent of colony formers in comparison to untreated controls. Earlier studies have tested some of the diCQAs against certain types of cancers. Phenolic extracts of YM that contained 5-diCQA and 3,5-diCQA along with other components, when tested on colon cancer cell line Caco-2, lung cancer A-549, esophageal cancer OE-33, and T24 bladder cancer, showed reduction in viability and proliferation rates of these cells [7]. YM phenolic extracts also inhibited SCC-61 and OSCC-3 squamous cell carcinoma cell lines [13]. Puangpraphant et al. tested methanol extracts containing a mixture of 3,4-diCQA and 3,5-diCQA as well as a fraction containing 4,5-diCQA on colon cancer cell lines CRL-2577 and HT-29. The authors surmised that the tested fraction inhibited cell proliferation through a caspase 3 activation and caspase 3 degradation pathway [8]. Our results of blockage of cell cycle progression in prostate cancer are distinct from that observed in colon cancer cells. This raises the possibility of 4,5-diCQA having different cellular targets in cancers of varied tissue origin. Use of a chemotherapeutic that works by a different mechanism, with 4,5-diCQA, should produce synergistic inhibitory effect on prostate cancer. It is also worth noting that studies so far reported in literature with YM extracts demonstrated no inhibitory activity on normal cells such as noncancerous CCD-18Co cell line [7] and fibroblasts [10]. The results from our studies with NIH-3T3 and MC-3T3 cells further confirmed the lack of toxicity on normal cells which would provide a higher therapeutic index for 4,5-diCQA on prostate cancer. Additional studies on the effect of 4,5-diCQA as well as its* in vivo* efficacy are warranted to determine the translational potential of this compound.

Effects on S-phase arrest coupled to Bcl-2 downregulation has been shown earlier to have antiproliferative effect in ovarian cancer cells [14]. Direct inhibition of Bcl-2 in head and neck associated cancers showed S phase arrest and inhibition of proliferation [15]. These studies corroborate with our observations with the DU-145 cells.

Future studies should explore the basis for the differential sensitivity of the various analogs, namely, 3,4-diCQA, 3,5-diCQA, and 4,5-diCQA. Similar to an earlier report [16], we also observed that the 4,5-diCQA was susceptible to degradation. Efforts are underway to stabilize the compound to attain better half-life which could potentially translate to enhanced* in vivo *efficacy. The findings with 4,5-diCQA if replicated through* in vivo *studies, either alone or in combination, could offer novel therapeutic strategies for prostate cancer therapy.

## Data Availability

All the data to make the conclusions is included in the article.
